# Beaming into the Rat World: Enabling Real-Time Interaction between Rat and Human Each at Their Own Scale

**DOI:** 10.1371/journal.pone.0048331

**Published:** 2012-10-31

**Authors:** Jean-Marie Normand, Maria V. Sanchez-Vives, Christian Waechter, Elias Giannopoulos, Bernhard Grosswindhager, Bernhard Spanlang, Christoph Guger, Gudrun Klinker, Mandayam A. Srinivasan, Mel Slater

**Affiliations:** 1 EVENT Lab, Faculty of Psychology, University of Barcelona, Spain; 2 Institució Catalana de Recerca i Estudis Avançats (ICREA), Barcelona, Spain; 3 Institut d’Investigacions Biomèdiques August Pi i Sunyer (IDIBAPS), Barcelona, Spain; 4 Fachbereich Informatik, Technische Universität München, Munich, Germany; 5 Guger Technologies (g.tec), Schiedlberg, Austria; 6 The Touch Lab, Research Laboratory of Electronics and Department of Mechanical Engineering, Massachusetts Institute of Technology, Cambridge, Massachusetts, United States of America; 7 Department of Computer Science, University College London, London, United Kingdom; Cajal Institute, Consejo Superior de Investigaciones Científicas, Spain

## Abstract

Immersive virtual reality (IVR) typically generates the illusion in participants that they are in the displayed virtual scene where they can experience and interact in events as if they were really happening. Teleoperator (TO) systems place people at a remote physical destination embodied as a robotic device, and where typically participants have the sensation of being at the destination, with the ability to interact with entities there. In this paper, we show how to combine IVR and TO to allow a new class of application. The participant in the IVR is represented in the destination by a physical robot (TO) and simultaneously the remote place and entities within it are represented to the participant in the IVR. Hence, the IVR participant has a normal virtual reality experience, but where his or her actions and behaviour control the remote robot and can therefore have physical consequences. Here, we show how such a system can be deployed to allow a human and a rat to operate together, but the human interacting with the rat on a human scale, and the rat interacting with the human on the rat scale. The human is represented in a rat arena by a small robot that is slaved to the human’s movements, whereas the tracked rat is represented to the human in the virtual reality by a humanoid avatar. We describe the system and also a study that was designed to test whether humans can successfully play a game with the rat. The results show that the system functioned well and that the humans were able to interact with the rat to fulfil the tasks of the game. This system opens up the possibility of new applications in the life sciences involving participant observation of and interaction with animals but at human scale.

## Introduction

The potential for immersive virtual reality remains largely untapped, and although the promise and excitement that it generated in the early 1990s has waned, it is an extremely powerful technology with applications that range far beyond those that have hitherto been developed. These have included simulation and training [1], therapy and rehabilitation [2], simulation of social situations in experimental studies [3], [4] and many others of a similar type. The vast majority of applications operate at human scale, except when virtual reality has been used for data visualisation, for example of data obtained from a confocal microscope [5] or for manipulation at the nanoscale [6]. Virtual reality still requires significant technical and conceptual advances [7] but such advances will come through novel applications that spur further technical and scientific research. In particular when combined with teleoperation it can open up a new class of applications such as the one considered in this paper.

Immersive virtual reality (IVR) and teleoperator (TO) systems provide the technical means for instantaneously transferring a person into a different place. An IVR system places people into a computer-generated environment where they can use their body normally for perception and interact with virtual objects, and with representations of other humans. Such virtual reality systems can be used to give people the illusion of being in the place depicted by the environment where they tend to behave as if what they were experiencing were real [8]. With TO an operator can have the sense of being physically in a remote real place, embodied there as a robot – seeing through the eyes of the robot whose actions are slaved to the motor actions of the operator. There the operator can, for example, operate remote machinery, collect samples, and so on.

When we combine IVR with TO we open up a new class of application where the human participant operates in a virtual (possibly transformed) representation of a remote physical space in which there are other live beings that may exist and act on an entirely different scale to humans. In particular here we show how to use IVR and TO to create a system that allows humans, and in principle, the smallest of animals or insects to interact together at the same scale. The fundamental idea is that the human participant is in an IVR system interacting with a virtual character (avatar) representing a remote animal. The animal is tracked in its physical space. The tracking information from the animal is relayed to the IVR and controls the actions of the avatar that represents it. The VR is scaled so that movements of the animals are mapped into appropriate changes in position of their avatar representations on a human scale. From the point of view of the humans there is a VR in which other live beings are represented with which they can interact.

We have so far described the setup from the human point of view - but how do the animals interact with the human, since the animals themselves are not in a virtual environment but in their own habitat without any special displays? The answer is that just as the animals are tracked and this information controls the movements of their virtual representations, so the humans are tracked and this controls the movements of a robotic device that is located within the animal habitat. Hence when the human, for example, moves close to the representation of the animal in the virtual environment, so the robot moves close to the corresponding animal in the physical habitat. There is a proportional mapping between the spatial relationships and orientations of the robot with respect to the animal in the physical space, and the human with respect to the animal’s avatar representation in the virtual reality. Both animals and humans experience their environment at their own scales. We call this process ‘beaming’ since the human in effect digitally beams a physical representation of him- or herself into the animal environment.

We describe an example of such a system that enables people to beam into a rat arena and interact with the rat at human scale, while the rat interacts with the human on the rat scale. In our particular application, a humanoid avatar represented the rat in virtual reality, and a small robot in the rat open arena represented the human. The human and rat played a game together as an example of the type of interaction that is straightforward to achieve in such a system. The purpose was to (a) Test the overall system performance during an interactive game played between person and rat. (b) To examine how the rat reacted to the robotic device. (c) To examine how the human participants accepted the setup and played the game, indeed whether it was possible to play the game at all.

**Figure 1:**
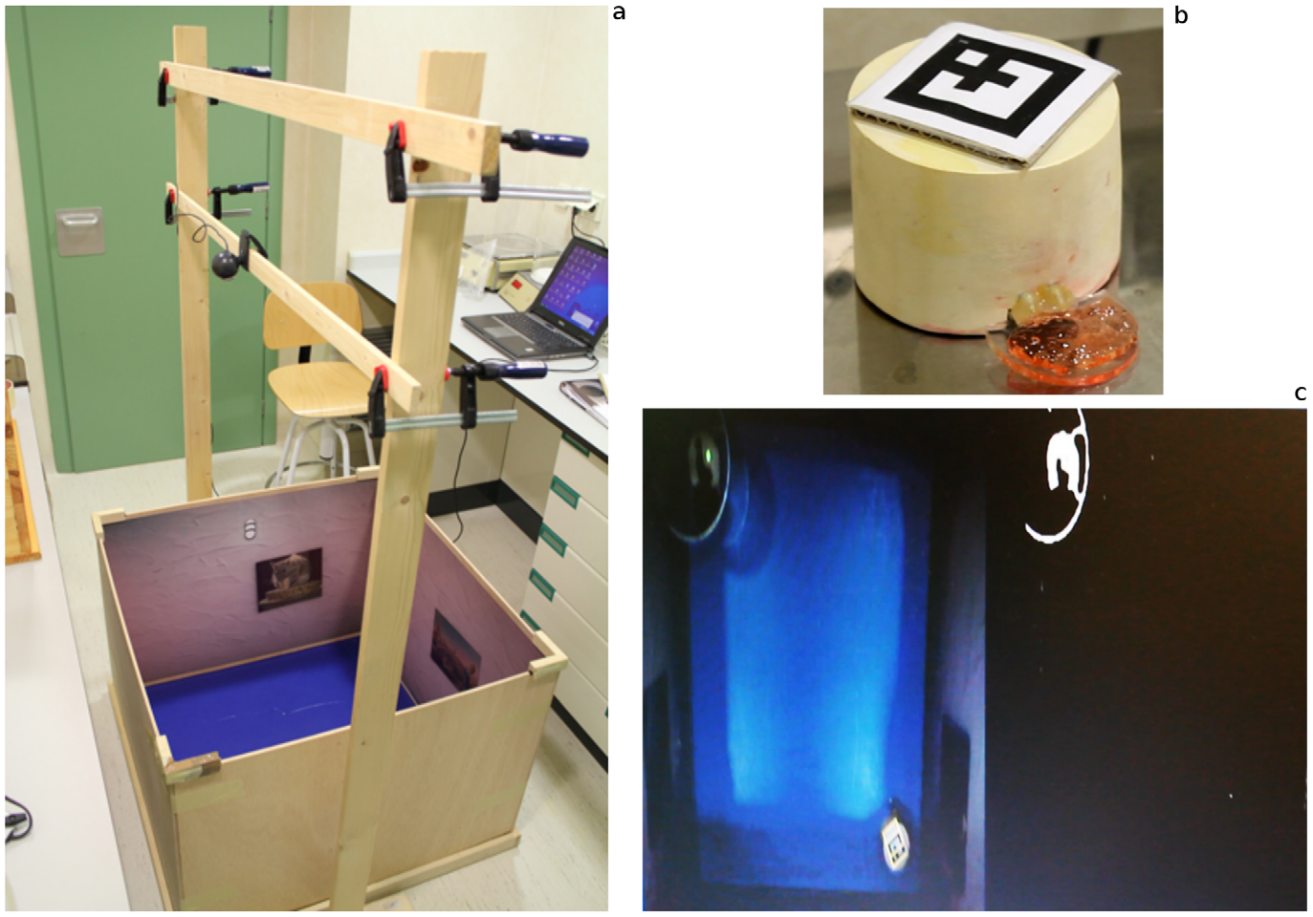
The rat arena and robot device. (a) Two of the pictures on the wall can be seen, and the frame on which a webcam was mounted for tracking purposes. (b) The e-puck robot protected by a purpose-made armour. For tracking purposes, a typical Augmented Reality marker was attached on top of the armour. The plastic platform in front was used to hold the food (strawberry jelly) for the rat. (c) Left hand side: View of the robot and rat for tracking. Right hand side: Result of the threshold used to detect the rat in the image.

## Materials and Methods

### Ethics Statement

The study was approved by the Ethics Committee of the Hospital Clinic (Barcelona, Spain) under the regulations of the Autonomous Government of Catalonia and following the guidelines of the European Communities Council (86/609/EEC). Participants gave written informed consent.

**Figure 2:**
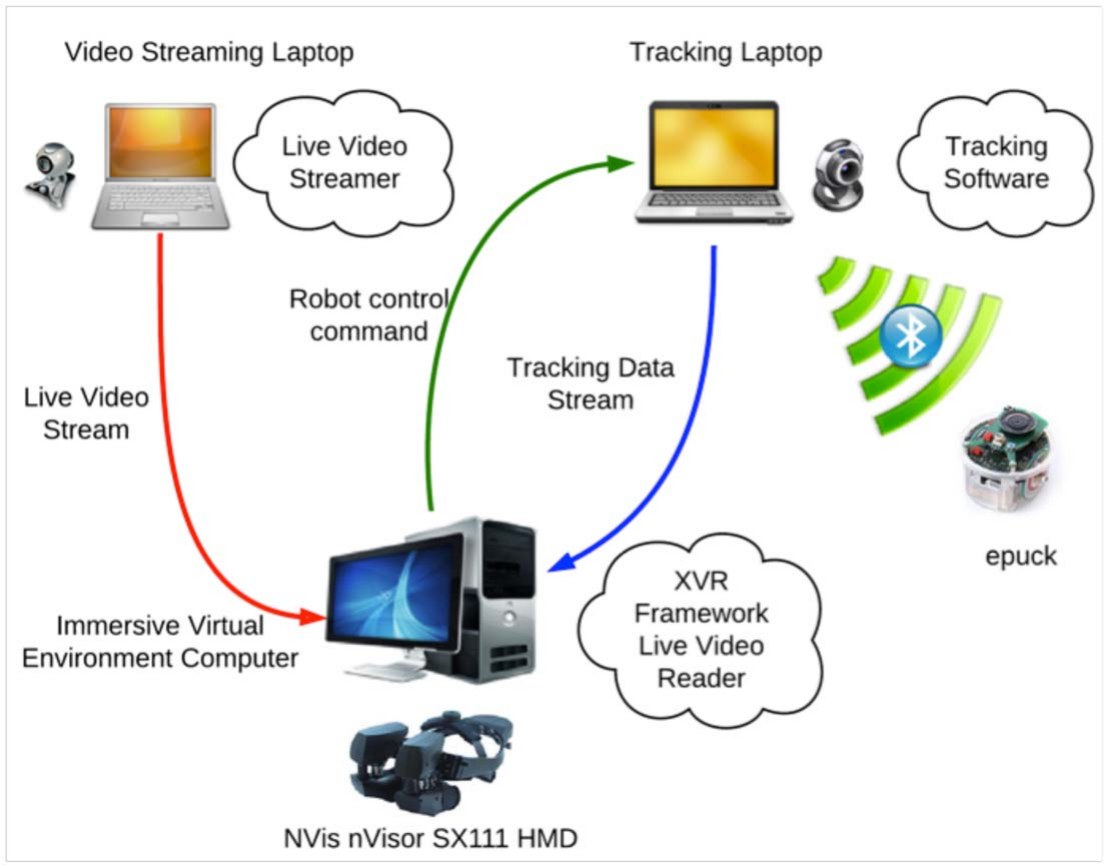
Simplified hardware and software architectures, and dataflow of the experiment.

### The Human-side Experimental Set up

A head-tracked wide field of view head-mounted display (HMD) was used. The HMD was a NVIS nVisor SX111 with a field of view of 76°×64° per eye, resulting in a total of 111°FOV and a resolution of 1280×1024 pixels per eye displayed at 60 Hz. Head tracking was performed by a 6-DOF Intersense IS-900 device.

**Figure 3:**
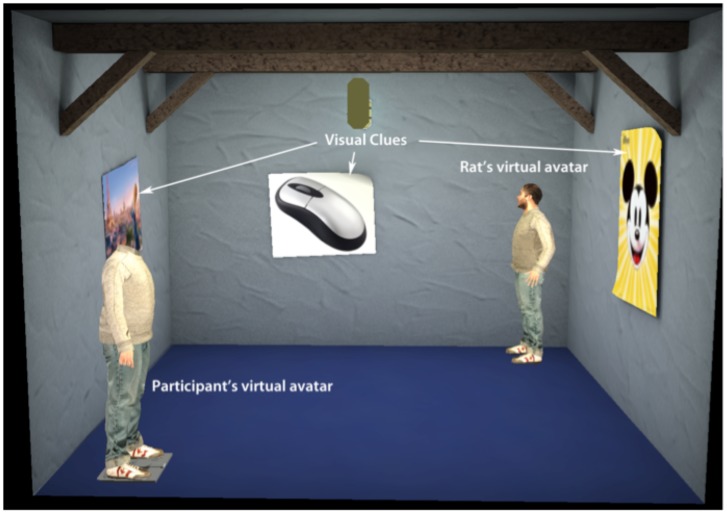
Screenshot of the virtual environment. Three of the four posters are visible in the image as well as the two avatars representing both the participant and the rat.

Due to the head-tracking, the participant could turn his or her head and body in any direction, and physically walk a pace or two. However, to move through the VR a hand held Intersense Wand was used. The participant could press a button on the Wand to move forward and backward at a speed constrained by the maximum speed of the robot in the rat arena. The rotation of the head tracker was used to change the direction of locomotion within the IVR and consequently of the robot’s movement.

**Figure 4:**
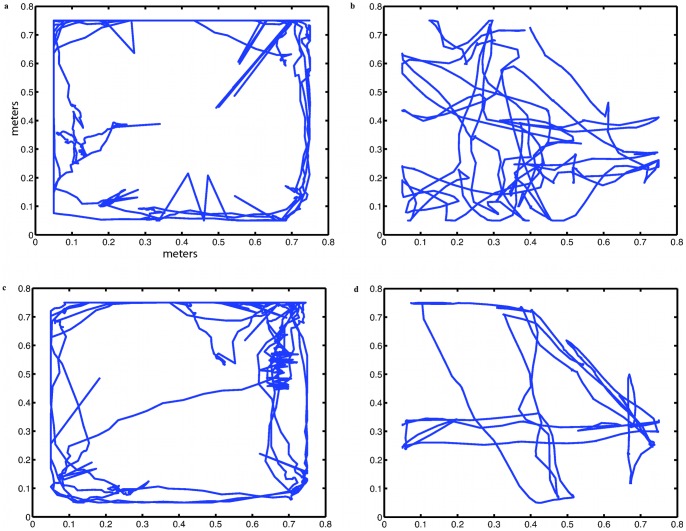
Movement of rats and humans (a) Rat A with (b) corresponding participant, (c) Rat B with (d) corresponding participant. Axes are in metres, and all movements are measured in the rat arena. Hence the human movements are those of the slaved robot.

### The Rat-side Experimental Set up

There was an open arena, a small robot and two webcams. The rat open arena was an 80 cm×80 cm×60 cm (width×length×height) box, with some pictures on the inside walls (Figure 1a). The rat was free to move anywhere in the box. Also inside the open arena was an e-puck® robot [9] (Figure 1b). The movements of the human in the VR were mapped to movements of this robot in real-time (Text S1). The e-puck has a size of 70 mm (diameter) by 50 mm (height), weighs 150 g and moves at a maximum speed of 12.9 cm/s. A small (65 mm×65 mm) marker was placed on top of the robot in order to facilitate camera based tracking of its position and to prevent potential errors due to the presence of the rat in the cage. Also the robot was encased in a special wooden handmade armour to avoid potential damage from the rat. The dimensions of the robot within the armour were 70 mm (height) and 90 mm (diameter of the armour). A food-support was attached to the armour in order to train the rat to follow the robot. The diameter with the food support was 120 mm.

**Figure 5:**
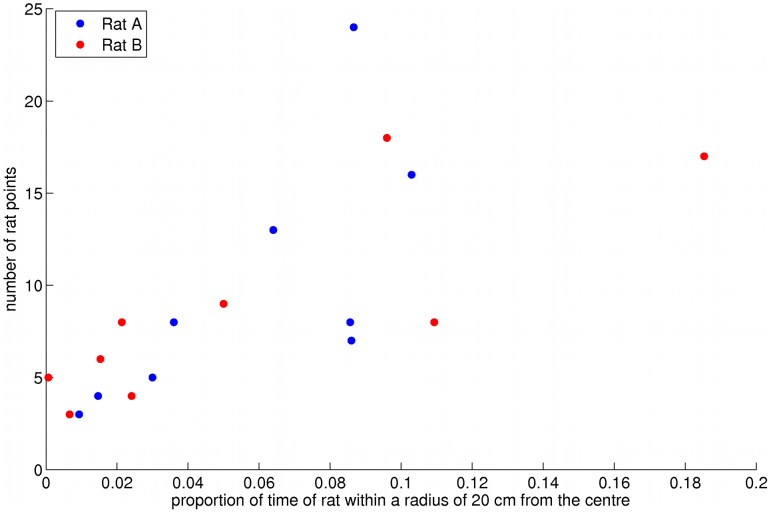
Scatter diagram of the proportion of time that the rat was within a radius of 20 cm from the arena centre by the number of rat points over all participants, for both rats. The number of rat points is the number of collisions between rat and robot that occurred away from the correct poster for the human to obtain a point. The Pearson correlation is significant for each rat separately (Rat A: r = 0.70, P<0.04; Rat B: r = 0.82, P<0.008).

Two webcams were mounted over the top of the open arena to do the tracking, from a top-view perspective looking down into the arena. The first one was used only for tracking (both rat and robot) while the second one was also used to convey video information to the human participant at various times in the course of the game. It should be noted that only one webcam would have been enough to perform both tracking and video streaming but with the drawback of high CPU usage on the computer.

### Overall Software Framework

Three computers were used each playing a different role, streaming different type of data (Figure 2). The three computers involved (two at the rat site and one at the human participant site), served the following functions:

- The first was dedicated to the tracking and control of the robot and tracking of the rat.
- The second was dedicated to video streaming from the rat open arena to the HMD machine.
- The third was dedicated to the management of the IVR (HMD display of the virtual environment and video from the rat site, tracking of the participant).

At the participant’s site, where the VR was displayed in the HMD, the software platform used was XVR [10]. XVR provided the framework to handle all the display and the networking activities related to streaming data, over the network arrangement of the various connected peers. The hardware accelerated library for character animation (HALCA) [11] was used for display and real-time animation of human characters.

**Figure 6:**
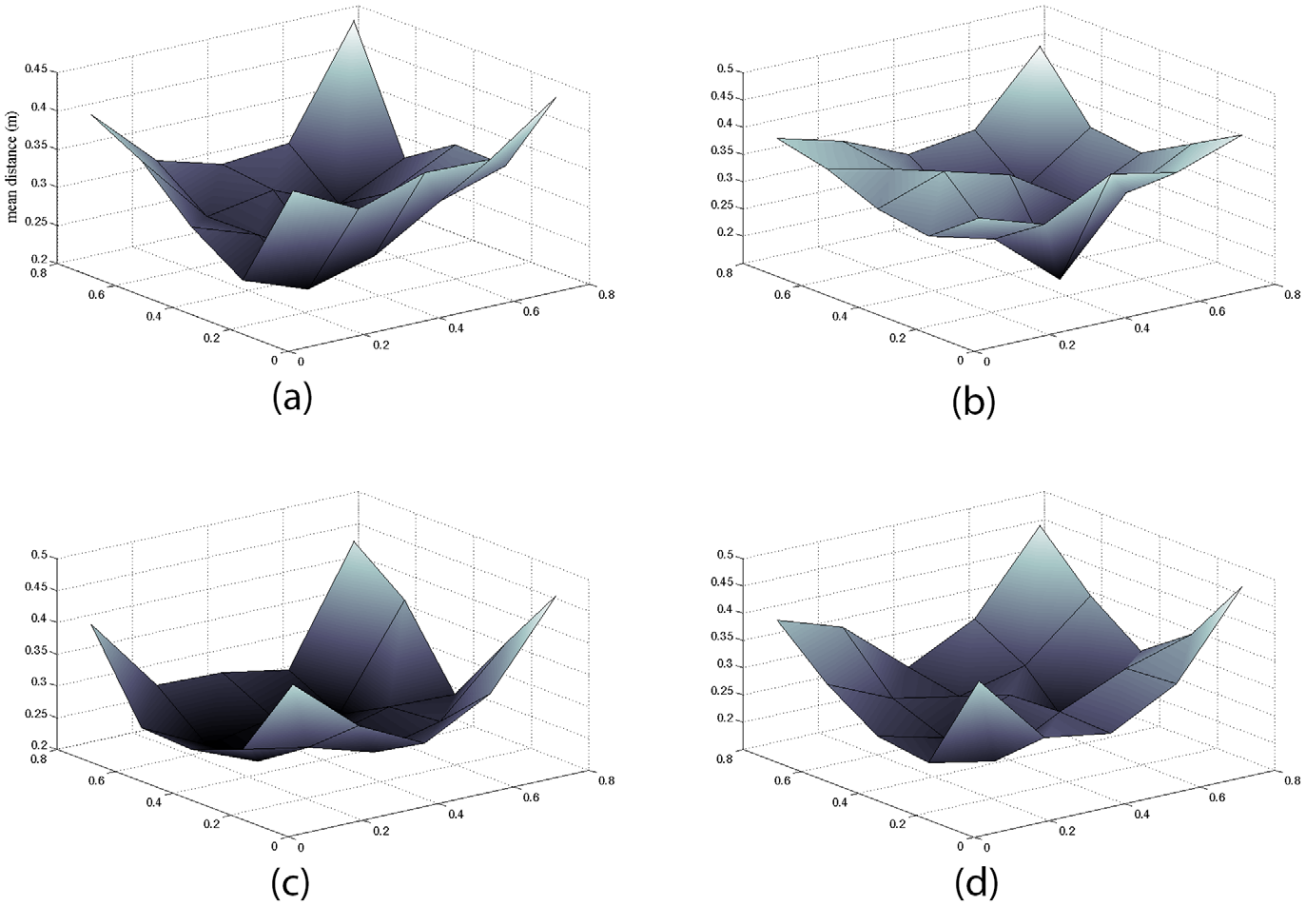
Distance between rat and robot by rat position. The vertical axis is the distance between the rat and robot corresponding to the position of the rat on the horizontal plane representing the rat arena. (a) Representing all 9 participants for rat A over trial 1 where the participants knew that the avatar represented a rat (b) The same participants for rat A over trial 2 where participants thought that the rat represented a remote human. (c) All 9 participants for rat B over trial 1. (d) The same participants over trial 2 for rat B.

At the rat site, the laptop dedicated to the tracking and robot control used MATLAB and Simulink (for the robot) and the Ubitrack framework [12] for the tracking. The second laptop was running the application dedicated to video streaming as well as a Skype chat where both experimenters (the one located on the rat site and the one located on the participant’s site) could keep in contact in order to ensure the smooth progress of the experiment.

**Figure 7:**
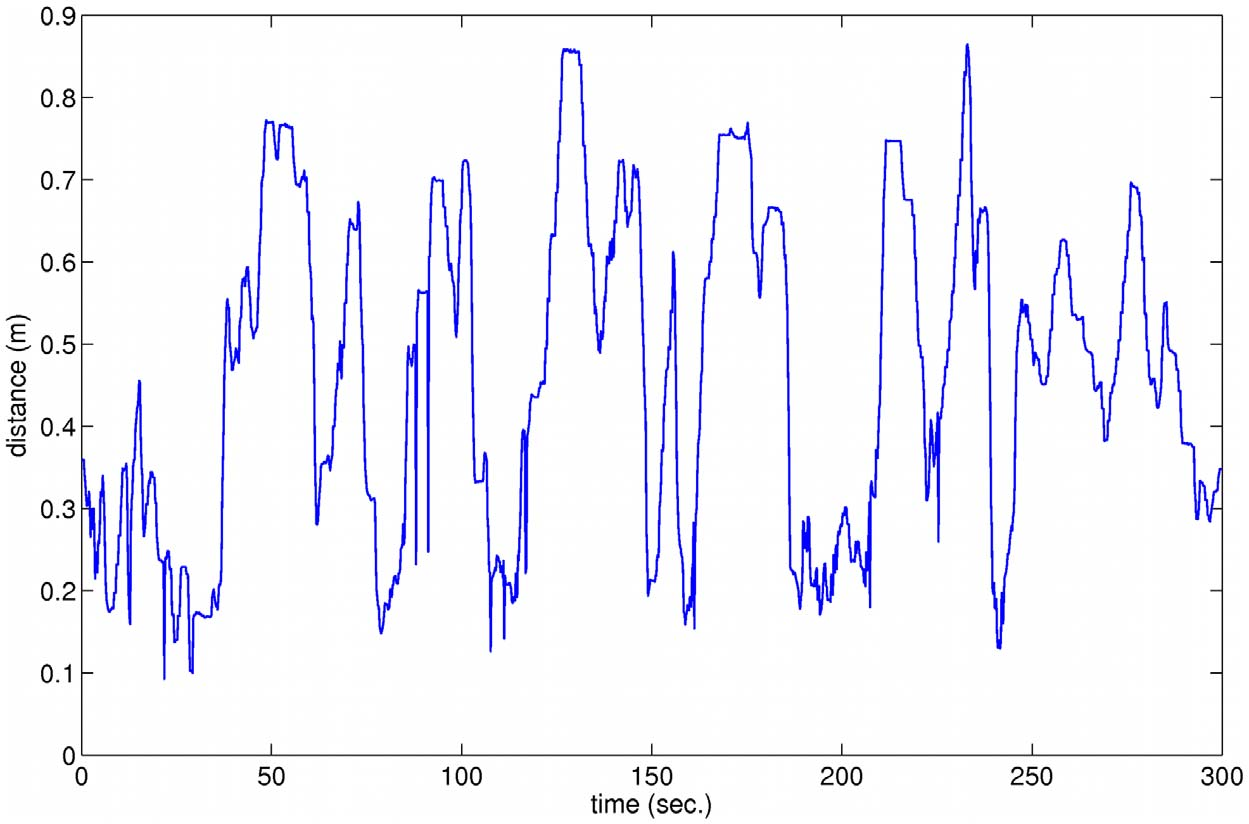
Waveform of distance between Rat A and the robot device for an arbitrary participant.

### The Virtual Reality

The VR displayed to the participant consisted of a closed 3D room with posters on the walls replicating the situation in the arena. The rat and the participant were each represented by an avatar (Figure 3) and were animated via the HALCA library. The XVR framework was used to display the VR stereoscopically to the participant in the HMD and to combine the various data flows (tracking, video, etc.) and devices together. The position of the avatar representing the rat was computed based on the tracking data received from the laptop located at the rat site. A walking animation was used to move this character from one position to another in order to maintain plausibility of the movements of the avatar. The participant controlled the position of his or her avatar by using head turns to orient and a button press on the Wand to move through the environment.

### Tracking in the Rat Arena

The rat and the robot in the open arena were tracked using a vision based tracking system. The system used a single camera mounted on top of the cage looking down into it, thus providing a bird’s-eye view. Two different tracking algorithms were implemented to estimate the trajectories and orientations of the rat and robot since they differed very much in their shape and behaviour.

Due to the cylindrical shape of the robot we were able to attach a typical rectangular, black-white pattern on its flat top surface. A marker-tracking algorithm, which is well researched in the computer vision community, was used to identify the position and orientation of the robot in three degrees of freedom each. The centre of the marker was associated with the centre of the robot since it was itself mounted in the centre. The orientation between the robot and the marker was estimated by a short registration procedure.

Two points on the rat were of interest: the major position being the body, and the subsidiary position the head for orientation. The first step in tracking made use of the already known position of the robot including its known extensions (i.e. the plastic platform used as food support) in order to exclude the space it occupied from the possible space of the rat. In order to estimate the rat’s body position the rat’s shape and outline are isolated in the current image through segmentation. The rat’s body position is then computed by searching for a global maximum of pixel intensities within its shape and outline.

Estimating the rat’s head position is slightly more complicated. Since the camera sees the rat from a top-view perspective, we could make use of the fact that the shape of the rat’s nose is triangular, and therefore relatively straightforward to detect. Once the nose position is known the rat’s head position can easily be estimated. As a consequence, a visual pattern matching approach was used to detect the rat’s nose position (rotated images of a rat’s nose were used as templates). The best matching position was chosen as the rat’s nose position and used to estimate the head position. In order to avoid jerkiness from one frame to another, an exponential moving average was applied to the head positions estimated in the current and previous frames.

The tracked body position of the rat was used to position the avatar in the virtual reality space, and the orientation was used to determine the forward-facing direction of the avatar. Although relatively simple, the methods to estimate the rat’s body and head positions proved to be efficient and robust.

Further technical aspects of the robot control, video and data streaming are discussed in Text S1.

### Interaction between Person and Rat

We tested our setup with a simple game that people could play with the rat. A video of all the phases is shown in Video S1. The participants entered the IVR through the HMD. They held the tracked Wand device in their dominant hand. There were two rats located in an animal care facility twelve kilometres distant from the IVR laboratory. Network communications between the two sites allowed sharing of the state of both the rat and the person, and therefore the computer programs were able to maintain the IVR and the physical environment in consistent states. The robot was slaved to the location and orientation of the tracked human. The rats had been earlier been trained to follow the robot, in order to get the food (jelly) on an attached tray (Text S1).

The participants were 7 men and 11 women from the campus (University of Barcelona). Their mean age was 23±2 (S.D.) years. They were non-experts in computer programming, had little or no experience with virtual reality, and were not much involved in computer game playing (Text S1).

Nine were assigned to one rat and the other 9 to the other rat. This was so that in one period of lab availability two participants could experience the system, one with one rat followed by the other with the second rat.

### The Scenario

The 80 cm×80 cm×60 cm (width×length×height) rat open arena had a different picture on each of its 4 walls (a computer mouse, the face of Mickey Mouse, a poster from the movie Ratatouille, a picture of a real rat with a piece of cheese, Figure 1a). The VR was a room of the same proportions as the cage, 3.2 m×3.2 m×3 m (width×length×height), and with the same pictures on the walls in the same places (Figure 3).

Upon arrival at the virtual reality laboratory the participant was given an information sheet to read that outlined procedures as part of the written informed consent process (see also Text S1 regarding the issue of excluding participants with animal phobia and further details of the procedures). Each session (completing paperwork, training and playing the game) took approximately 30 minutes, and the participants were paid 10€ for their time.

Then participants donned the HMD and held the Wand in their dominant hand and were instructed to look around the scene and describe what they saw. There was then a training period where they learned to navigate the environment using the Wand. Then in the remote animal care facility, the rat and robot were placed into the cage, and the whole system was started (rat tracking, robot activation and tracking and display) and the participant would then see the avatar representing the rat in the IVR. In order for the participants to understand that they were actually interacting with a remote rat, and the relationship between their own movements in the IVR and the robot movements in the rat arena, the experimenter switched, several times, the view in the HMD between the VR and a bird’s-eye video stream of the rat cage containing the rat and the robot device. Finally a simple procedure was carried out to convince the participants that what they were seeing in the video of the rat arena was live and that the VR represented this (Text S1).

The interaction between the rat and the person was designed as a game that lasted for 5 minutes. The participants were told that they would win a point when they were situated close enough to their opponent avatar provided that they were standing by the ‘correct’ poster at the time, and that success would be signified by a bell ring. The game was played in a series of rounds and at each round the point-winning poster was changed, but the participant was not informed about which was the correct poster except for the very first one. They were told that they would lose a point to the opponent (signified by a horn sound) whenever they were close to the avatar but situated anywhere except under the correct poster. The purpose of this was to encourage the participant to move around the virtual room and to engage their opponent avatar.

The minimum distance between rat and robot in order for the human to gain a point was set to 10 cm in the rat open arena coordinates. This threshold was motivated by the size of the armour encompassing the robot and the imprecision of the rat position due to the tracking. The minimum distance between the participant and the correct poster on the wall was set to 28 cm.

Two such games were played by each person. In the second game participants were in the same virtual room with the virtual character. However, this time the switch to the video view showed a woman waving at them (a bird’s eye view from approximately 4 meters high) and near her was a small humanoid robot. It was explained that everything was the same as before, except that now their opponent was a remote human, and that the humanoid robot that they could see was their own representation. In reality this video had been pre-recorded, there was no remote human participant, and during this second phase of the experiment the rat again controlled the avatar in the virtual environment. The purpose of this second trial of the experiment was only out of interest to see whether the behaviour or attitudes of the participants changed when their opponent was believed to be the rat compared to when it was believed to be human. This second game lasted also 5 minutes under the same conditions as the previous one. After removing the HMD, they were interviewed, debriefed about the purpose of the experiment, and paid.

## Results

### System Performance

A number of measures were used in order to evaluate the performance of the system, in terms of network performance, video streaming latency and robot command latency. The software architecture of the experiment was distributed on three different machines at the two different physical sites both connected via the internal network of the University of Barcelona (Figure 2). Hence, a ‘ping’ command issued between distant computers, which corresponds to measuring the time between sending and receiving back 32 bytes of data, showed an unnoticeable delay (<1 ms). The video stream required sending a 640×480 pixels RGB video between two distant computers. The latency measured revealed a delay of 120 ms (±20 ms) between a frame sent from the video streaming laptop and the IVR computer. Finally, the measured delay of the robot command stream between the computer responsible for tracking and that running the virtual reality displays was 150 ms (±20 ms). This delay corresponded to sending a command via the UDP protocol from the IVR computer, receiving this command on the tracking computer in the MATLAB software, and processing the command before finally sending it to the robot via the Bluetooth protocol. The Bluetooth protocol itself induced a delay up to 20 ms. The human participants in virtual space and the rat and robot device in the physical space of the open arena were tracked at the sampling rate of 30 Hz.

Since there is no Gold Standard algorithm against which we can compare the accuracy of our system we only can provide the algorithm’s runtime, which was estimated as 10 ms for the calculation of the rat's major position and 20 ms for the estimation of the head position and viewing direction on an Intel Core2 Duo CPU with 2.50 GHz. The robot tracking which is marker-based is very efficient and is negligible compared to the rat tracking.

Putting everything together the time spent in the tracking process represents roughly 30 ms, which consists of both robot and rat tracking (body position, head position and head orientation).

### Movement Distributions

The two rats both showed typical navigational patterns, staying close to the walls for most of the time, with occasional forays towards the centre. This is a typical behaviour of rodents referred to as thigmotaxis, enhanced by illumination [13] which was the case in our experiments. Figure 4 shows movements over the whole period of an arbitrarily selected trial for both rats, and the movements of the corresponding participants. It is shown that the rat tended to gravitate towards the edges and corners. The human covered more the central area to entice the rat towards the centres of the walls (where the posters were located).

Rats were trained to follow the robot in the search for reward, and thus the principal reason for the rat to move away from the thigmotactic pattern of remaining close to walls and corners was most probably the presence of the robot. This can be seen in Video S2, which shows 6 typical sequences of the movements of rat and robot.

We obtained all of the (x, y) positions of each of the two rats during all the trials using the sampling unit of time as 0.2 s following [14]. The proportion of time that the tracked centre of the rat’s body (without tail) was within a radius of 20 cm of the centre of the arena was computed. The rats were approximately 18–20 cm in length and 5–6 cm in width. Hence a radius of 20 cm in the area size of 80×80 cm^2^ indicates a region quite distant from the edges. We counted the number of times that there was contact between the rat and robot that occurred while not by the correct poster, referred to as ‘rat points’ (since the humans only obtained a point when the collision was near the correct poster). Figure 5 shows the number of rat points by the proportion of time that the rat was in this central region, over all participants and for both rats (for the first trials only). There is a linear relationship between these (Pearson r = 0.71, P<0.001) indicating that the greater the time that the rat was in the centre the greater the number of collisions with the robot. Since the participants knew that they would lose a point in the game if a collision occurred that was not under a poster, it is likely that such encounters were due to the rat following the robot, rather than through the actions of the human. A similar result holds for a radius of 15 cm, and even with a radius of 10 cm the relationship is still significant for rat A (r = 0.89, P<0.0015).

Was the game played? Corresponding to each (x, y) position was the distance between the robot and the rat at that moment (which itself was directly proportional to the distance between human participant and the avatar representing the rat in the VR). We divided the arena floor into a 5×5 grid and found the mean distance between rat and robot for each grid cell over all the participants. We were interested to see whether any pattern could be found that indicated that movements were not just random, and that indeed the game was played. Figure 6 shows the resulting graphs.

The figure shows that the distance between rat and robot (human) was greatest when the rat was in its starting corner or an adjacent corner. The graphs also show minima where the posters were located indicating that the game was being successfully played. This is most pronounced in the case of Figure 6 (a) and least pronounced for Figure 6 (b) which corresponded to trials when the participants believed that they were playing against a human opponent. However, in almost all cases the mean distances near the posters are significantly less than the mean overall distance between the rat and robot taken over the whole time period. This can be shown by calculating the normal z-statistic for comparison of a sample mean with a population mean, here taking the population mean to be the mean distance over the whole time period for a particular rat and trial. These overall means are 0.37 m and 0.39 m for Rat A for trials 1 and 2 respectively, and 0.38 m and 0.40 m in the case of Rat B. For Rat A in trial 1 the four regions in the 5×5 grid corresponding to the positions of the posters have |z| >4 for all but one, and similarly for trial 2 all |z| >3.6 except for (the same) one. For Rat B all |z| >6.6 for trial 1, and all |z| >10.7 for trial 2. This does strongly suggest that the distances around the posters were usually quite different from the overall distance.

The time varying distance between the rat and the robot representing the human is illustrated in Figure 7 which shows the plot of distance between the rat and the robot (human participant) over the 5 minute period of the experimental trial, following the same rat (A) and participant as in Figure 4 (a, b). This is typical of all such plots representing the dynamics of movement of both rat and human, as they approached each other and moved away again. The evidence suggests the distances between rat and human tended to be slightly greater in trial 2 than in trial 1. In trial 2 participants believed that their opponent was a human. This change in distance could be due to that belief and therefore the desire of human participants to follow rules of proxemics, that is to keep a socially acceptable distance from their opponent, or could be due to the fact that in the second trial the game was played less successfully than in the first. In fact the total number of points scored by participants in the second trial was about half that scored in the first trial. This may have been because the rats were tired or satiated, or it could have been because the humans believed that they were playing against another real human, and adjusted their behaviour accordingly. The evidence regarding this issue is considered and weighed in Text S4.

## Discussion

Since this is a newly developed system it is interesting to consider possible applications. Unlike existing ethological studies of animals, for example, cats [15] and horses [16], it may be interesting for life science investigators to obtain an entirely different view of animal behaviour, by seeing the animals on a human scale, even represented as humans. This would offer a possibility of participant-observational study of animal behaviour and generally of animal communities in a way never before possible. Such changes of view may offer quite new insights.

It might be thought that generally rats would not behave normally when there are robots in their vicinity. However, the placing of robots in rat arenas has been carried out before, as part of the quest to develop a robot that is rat-like in its behaviour. For example, in one system [17] a robot that emulated some rat-like behaviour was placed in a open arena with a rat. An experimental study concluded that the robot influenced the rat behaviour in an appropriate way. Ultimately the authors wished to create robots that would interact with humans; however, working with rats provided an environment in which to understand the relationships that may develop between animal and robot in a simplified form. Other work has also had this motivation [18], where the rat and robot developed a symbiotic relationship over many hours, and where the robot could learn to manipulate the behaviour of the rat.

Generally there is an increasing amount of work that seeks to understand animal behaviour for the engineering of robots and then testing the robots in the context of interacting with the animals that they emulate, for example, an ‘animat’, a robot that navigates like a rat [19]. The flow of understanding is two-way, where such animal-based robots can shed light on animal behaviour and cognition.

To our knowledge there has never been a system where a physical device operating in a rat environment acts as a surrogate representation of a human operating in an equivalent virtual environment. Some specific computational requirements are discussed in Text S2, but in general the system components needed to do this are: (a) An IVR system that can track the movements of a human participant; (b) A device that can be slaved to the actions of the human which is located in the animal space - a teleoperation system; (c) Tracking of the animals in their space and the relaying of the tracking information to control avatars in the virtual environment; (d) A network capable of real-time distribution of data between the human and animal sites; (e) A virtual model of the remote (animal) locale. As an example, this type of system could even be used to allow interaction between humans and birds or flying insects. There exist today flying robots [20] so that (b) would be supported. Moreover, it is possible to track, for example, birds [21] so that (c) would also be supported. Also in relation to (b) another instance of this type of system could replace the robotic device by a real rat with its movements controlled remotely though brain stimulation [22].

In the paragraph above we have extended beyond a single animal - which requires the capability to track multiple animals simultaneously and thereby control multiple avatars. Moreover, the same could be extended to multiple human participants (further technical details are discussed in Text S3). Virtual reality has previously been used for communication between multiple participants where people in remote places can meet in a virtual environment shared by all. In such applications each of the participants uses their own virtual reality system, perhaps separated by thousands of kilometres, and they can see and talk to life-sized representations of one another, and carry out tasks together [23]. This is facilitated by Internet network protocols that distribute the data between the various systems, and each system is responsible for displaying the virtual environment from the viewpoint of its particular participant. This has even been achieved with haptic interaction between the remote participants [24], [25]. However, what is different in our system is that the human is represented in the animal environment through a physical surrogate. In shared virtual environments all participants are in a virtual reality system. In our case only the human is in such a system, whereas the animals are located in their own physical environment without any need for virtual displays.

The conjunction of immersive virtual reality with teleoperator systems supports a class of application that would be very hard to achieve through any other means. The virtual environment acts as a unifying medium through which participants who operate at quite different scales can be brought together, and their appearance changed as appropriate to the demands of the application. Although we have applied this technology to interaction between humans and animals, primarily for use in the life sciences, the very same idea could be used for example, to realise human to remote-human interaction, with an example of such remote communication described in [26], [27].

## Supporting Information

Text S1 **Supporting procedures and methods.** A number of procedures and methods are described in detail, including rat training, robot control, video and data streaming, and experimental procedures.
(DOCX)

Text S2 **Computational and network requirements.** This describes the technical computational requirements to execute the system described.
(DOCX)

Text S3 **Multiple participants and animals.** This describes what would be needed to extend the system to cater for multiple human and animal participants.
(DOCX)

Text S4 **Distance distributions in trials 1 and 2.** This presents further analysis of the distances between the rat and human participants, and in particular there is a comparison between trials 1 and 2.
(DOCX)

Video S1 **A human participant interacts with the rat represented as a virtual human character in immersive virtual reality.**
(MP4)

Video S2 **The first 200 seconds of rat and robot movements for 6 participant trials.** The rat is represented by the blue square and path, and the human is represented by the red circle and path. Note that the sizes of the square representing the rat and the circle representing the robot are much smaller than would be if they were drawn to scale. Hence the videos under-represent the closeness of the rat and robot. The video timing is not real-time.
(MP4)
