# Supplementary material for: Beaming into the Rat World: Enabling Real-Time Interaction between Rat and Human Each at Their Own Scale
Source: PLoS One. 2012 Oct 31;7(10):e48331. doi: 10.1371/journal.pone.0048331 (PMC3485138; doi:10.1371/journal.pone.0048331)
Supplement: Text S1 — Supporting procedures and methods. A number of procedures and methods are described in detail, including rat training, robot control, video and data streaming, and experimental procedures. (DOCX) [file pone.0048331.s001.docx]

Supporting Text S1

Beaming into the Rat World: Interaction Between Rat and Human Through an Immersive Virtual Environment

Jean-Marie Normand^1^, Maria V. Sanchez-Vives^2,3^, Christian Waechter^4^,
Elias Giannopoulos^1^, Bernhard Grosswindhager^5^, Bernhard Spanlang^1^,
Christoph Guger^5^, Gudrun Klinker^4^, Mandayam A. Srinivasan^6,7^, Mel Slater*^1,2,7^

^1^EVENT Lab, Faculty of Psychology, University of Barcelona, Spain

^2^Institució Catalana de Recerca i Estudis Avançats (ICREA), Barcelona, Spain

^3^Institut d’Investigacions Biomèdiques August Pi i Sunyer (IDIBAPS), Barcelona, Spain

^4^Fachbereich Informatik, Technische Universität München, Germany

^5^Guger Technologies (g.tec), Austria

^6^The Touch Lab, Research Laboratory of Electronics and Department of Mechanical Engineering, Massachusetts Institute of Technology, Cambridge, USA

^7^Department of Computer Science, University College London, UK

Corresponding Author:

*Mel Slater

ICREA (Institucio Catalana de Recerca i Estudis Avançats), Barcelona, Spain

melslater@ub.edu

# Rat training and behaviour

Two adult Lister Hooded rats were trained to be in the same arena with the robot and to follow it. Rats were cared for and treated in accordance with the Spanish regulatory laws (BOE 256; 25-10-1990).

All experiments were approved by the Ethics Committee of the Hospital Clinic (Barcelona, Spain) under the regulations of the Autonomous Government of Catalonia and following the guidelines of the European Communities Council (86/609/EEC).

We did not require any particular behaviour from the rats, but it was necessary to avoid them sitting passively in a corner, given that some interaction with the robot was necessary for the experiment. During the session the rats were placed in the arena described above. The rats were previously trained to follow a toy car that had a platform of 3 cm diameter containing jelly. The rats would follow the car to eat the jelly. The robot had a small tray attached to hold some jelly, and following the training the robot was introduced in the arena, and the rats became familiar with it and followed it in search for the jelly.

# Robot control

The e-puck was controlled via a Simulink component in the MATLAB software constructed for the purposes of this experiment which provided a way to control it through rotation angle (between 0° and 360°) and speed (normalized between 0 and 1000). An angle of 0° meant that the e-puck would go straight while an angle of 180° would make the robot move backwards. A rotation of the robot on its left hand side was represented by an angle between 1° and 179° while a rotation on the right hand side was represented by an angle between 181° and 359°. A speed of 0 represented no movement while a speed of 1000 represented the full speed of the robot (approximately 13cm/s). As a consequence in order to control the e-puck robot a UDP packet was sent representing two values, namely ‘angle’ and ‘speed’ to the tracking laptop. In order to compute those values the 2D position [X_p_ ,Y_p_] of the participant in the immersive virtual reality (IVR) scene was converted into their open arena space [X_c_ ,Y_c_] counterparts. Those open arena coordinates represented the position where the robot should be in order to represent the position of the participant.

The tracking system provided the current 2D position of the robot [X_r_ , Y_r_] in the open arena coordinates. Based on the vector between [X_c_ , Y_c_] and [X_r_ , Y_r_], the angle (as described above) corresponding to the correct displacement of the robot was computed. At any moment the speed of the robot was set to either 0 or 1000.

The connexion between the robot and MATLAB was made via UDP packets encapsulated in the Bluetooth protocol. According to Gctronic, the company selling the e-puck, the Bluetooth connection involves a delay between 3ms and 20ms between sending a command from MATLAB and receiving it on the robot. Due to the delay in the control of the robot induced by network transfers (principally due to the Bluetooth protocol), and the imprecision between the coordinates of the participant in the IVE and of the robot in the cage, we had to introduce a ‘safe area’ in which the position of the robot was considered correct. This area was modelled as a circular zone of 3cm diameter. The choice of 3cm was made by measuring the size of the food support, which increased the size of the armour by the same amount. For example suppose the target position of the robot in the open arena was [75.2 , 48.7] and its current position [73.7 , 47.3]. The distance between those two points is 2.05cm, and since this is less than 3cm the position was considered correct and the robot would not move. This distance was set empirically in order to prevent the robot from doing small back and forth movements when the participant did not move in the virtual reality.

# Video streaming

In order to stream video from the rat site to the participant site, a dedicated application was developed in C++ with the OpenCV and the FFmpeg libraries. Each frame from the webcam was captured with the OpenCV library, then converted into an OpenGL compatible data structure supported by the FFmpeg library before being streamed to the computer on the participant site. Once the frame was received it was converted back into an OpenGL texture so that it could be displayed in the XVR platform.

# Data Streaming

In order to update the positions of the avatar representing the rat (in the virtual environment) and the robot (in the cage), data was exchanged between the IVR computer and the tracking laptop. Indeed, based on the tracking data (2D positions of the rat and the robot) from the laptop the new position was computed and used to update the animation of the avatar representing the rat in the IVE and to create the command sent to the robot controller based on the current position of the robot and the position of the participant’s avatars. The exchange of this data was implemented with UDP packets between the tracking laptop and the computer in charge of the virtual environment. The tracking software sent 2D positions of the rat and the robot to the IVE computer while the computer sent back to the Simulink program on the tracking laptop the command the robot had to perform.

# Participant Background

The 18 participants answered a questionnaire prior to the experiment that gave information about their experience with computers, virtual reality and computer game playing. On a scale of 1 to 7 where 1 meant no experience of computer programming and 7 meant expert, the median score was 2 and the interquartile range was 3. Regarding prior experience of virtual reality on a scale of 1 (none) to 7 (expert) the median and interquartile ranges were both 1. The extent to which these participants played computer games was also low. On a question where 1 meant that they never played computer games in a typical week and 7 meant more than 9 hours a week the median and interquartile range were both 2.

# Checking for Animal Phobia

A short questionnaire was given to the participants before their trials in order to check that they did not have any kind of animal and especially rat-related phobia. This did not occur, but we had planned that such participants who could not take part in the experiment would be shown some other virtual reality environment in compensation. Participants were asked whether they wished to continue after the experiment had been outlined to them and if so they completed a consent form, followed by a questionnaire that gathered basic information about their age, prior computer knowledge, prior experience with virtual reality, and computer game playing.

# Procedures

Once participants had entered the virtual environment they were asked to look around the scene and also to look down towards themselves and they would see a virtual body substituting their own. They were then instructed on how to move around the virtual room by using the Wand – looking in the direction they wished to move while pressing a button on the Wand would move them forward in that direction. They had a training session where they were asked to move towards a series of flashing cues that were presented sequentially. When the participant had managed to find and approach each of these nine cues the training was complete.

After this, the participants were instructed to close their eyes. Meanwhile, in the remote animal care facility, the rat and robot were placed into the cage, and the whole system was started (rat tracking, robot activation and tracking and display – see Methods). Upon opening their eyes, the participants were asked what was different about the scene, and they all answered that they could now see someone else (the avatar) there. They were asked to move around the scene and to go towards the other character. After they had changed their position a few times in the room, approaching towards and moving away from the avatar, the display was switched to show a video bird’s eye view from the top of the rat arena. Now participants were again asked to describe what they saw, and all eventually reported that they could see the rat. They were prompted to say what else they saw - in particular we wanted them to notice that the pictures arranged on the arena walls were the same as the ones they could see in the virtual room. Also they were asked to look for the robot, which was seen by them from above as a white circle. They were asked to move by pressing the Wand button as they had already learned, so that they could see that when they moved themselves in the virtual environment so the robot moved in the rat cage. Several times the experimenter switched between the virtual reality and the video view, so that participants could learn that the spatial relationships between the robot and the rat and posters in the room were mapped to the relationships between themselves in the virtual reality, the other avatar, and the posters in the virtual room.

Finally after this training period the virtual reality HMD screens went black for a moment, and when the scene reappeared the game started and lasted for 5 minutes. At the end of the game the screens went black again, and some questions were read to the participants from a short questionnaire.

# Demonstrating that the rat video was live

In the many pilots that we had carried out in preparation for this study we discovered that the vast majority of participants found it very difficult to comprehend what was happening, and although at some abstract level they would understand that they were controlling the device in the rat cage, they thought that this was also some kind of virtual reality that was not really happening. In order to try to overcome this problem the experimenter at the site of the rat arena wrote out on a piece of card in large lettering: “Today is [*today’s date*] it is now [hour and minutes]. Hello [*name of the participant*]! Thanks for your participation.” This was placed under the open arena camera so that the participants would see it and was followed by a hand waving from the experimenter in the animal care facility. This was to try to convince the participant that these events were really happening live, that there really was a rat in a distant open arena with which they were interacting.
