# Supplementary material for: Beaming into the Rat World: Enabling Real-Time Interaction between Rat and Human Each at Their Own Scale
Source: PLoS One. 2012 Oct 31;7(10):e48331. doi: 10.1371/journal.pone.0048331 (PMC3485138; doi:10.1371/journal.pone.0048331)
Supplement: Text S2 — Computational and network requirements. This describes the technical computational requirements to execute the system described. (DOCX) [file pone.0048331.s002.docx]

Supporting Text S2

Beaming into the Rat World: Interaction Between Rat and Human Through an Immersive Virtual Environment

Jean-Marie Normand^1^, Maria V. Sanchez-Vives^2,3^, Christian Waechter^4^,
Elias Giannopoulos^1^, Bernhard Grosswindhager^5^, Bernhard Spanlang^1^,
Christoph Guger^5^, Gudrun Klinker^4^, Mandayam A. Srinivasan^6,7^, Mel Slater*^1,2,7^

^1^EVENT Lab, Faculty of Psychology, University of Barcelona, Spain

^2^Institució Catalana de Recerca i Estudis Avançats (ICREA), Barcelona, Spain

^3^Institut d’Investigacions Biomèdiques August Pi i Sunyer (IDIBAPS), Barcelona, Spain

^4^Fachbereich Informatik, Technische Universität München, Germany

^5^Guger Technologies (g.tec), Austria

^6^The Touch Lab, Research Laboratory of Electronics and Department of Mechanical Engineering, Massachusetts Institute of Technology, Cambridge, USA

^7^Department of Computer Science, University College London, UK

Corresponding Author:

*Mel Slater

ICREA (Institucio Catalana de Recerca i Estudis Avançats), Barcelona, Spain

melslater@ub.edu

# Computational and Network Requirements

In the experimental setup (rat-side) we used 2 (relatively old) laptops (CPU: Intel Mobile Core 2 Duo T7200@2.00 GHz – RAM: 2.0 GB – GPU: NVIDIA Quadro FX 350M with 256 MB RAM) in order to lessen the computational cost of running MATLAB (to control the e-puck), the tracking software and the video stream at the same time. Of course at least the same system performance could be achieved using a more recent classical desktop computer or a powerful laptop, that is a multi-core CPU (>= 4) and a large amount of RAM memory (>= 4 GB). This would allow each of the processes to run on its own core and ensure real-time capabilities.

On the human-side of the experimental set-up, as for any immersive virtual reality application, we require a powerful desktop computer with high-end graphics capabilities (CPU: Intel Core 2 Quad Q9550@2.83GHz – RAM: 4.0 GB GPU: NVIDIA GeForce GTX 480 with 1.5 GB RAM). Indeed, the computer must be powerful enough to run the VR environment at least at 60 frames per second in order to ensure smooth 3D for the participant.

In terms of network communications, we used the University of Barcelona’s internal network ensuring optimal performance for our data exchange and to ensure that we would not lose the connection during the experiment. Nevertheless, we believe this experiment could be run over a classical wired Internet connection (e.g. 10 Mbits/s) provided that the video streaming would be optimized. The amount of data transferred (apart from the video) is very small and requires less network capabilities than a classical online game (e.g. a massively multiplayer role playing game).

Concerning the video stream, the frames could be compressed before being send over the network in order to minimize the size of data exchanged over the network. Of course, image compression should then be performed both by the sender and the receiver of the images and would increase the computational requirement, but we believe that one high-end computer would be sufficient at both ends of the experimental setup (human and rat) in order to perform this experiment.

Data streaming could also be possible over a wireless connection (Wi-Fi) provided some reliable data transmission mechanism is implemented (in particular UDP packets do not offer reliable service and can disappear or get lost in the transfer). This could add some delay in tracking positions exchange which accumulated with the Bluetooth protocol delay, while communicating with the e-puck, might not be desirable.

Regarding the length of continuous operation: the e-puck has a 2-hour autonomy while moving, but of course the main constraint of continuous operation would be because of the rats. We would recommend resetting the Bluetooth communication between MATLAB and the e-puck after each trial in order to clear the Bluetooth stack.

Apart from the robot autonomy and ethical requirements for the animals, there is no technical limitation to the duration of such an experiment provided that the software is designed to support this (i.e., a scenario that can run for a longer time).

Finally, setting up the ‘rat site’ of the experiment took a single person about 30 minutes. This consisted of:

- Putting on special clothing for safety/cleanliness issues;
- Assembling the rat cage;
- Assembling the mounts that hold the cameras (for tracking and video streaming);
- Booting the computers and setting up the software;
- Calibrating the tracking system;
- Preparing the robot, connecting it to the computer, checking the connection and putting jelly onto it;
- Dealing with the rats;
- Testing everything before the experiment, especially the connections with the tracking system and the robot and with the experimenter at the ‘human site’.
