# Supplementary material for: Beaming into the Rat World: Enabling Real-Time Interaction between Rat and Human Each at Their Own Scale
Source: PLoS One. 2012 Oct 31;7(10):e48331. doi: 10.1371/journal.pone.0048331 (PMC3485138; doi:10.1371/journal.pone.0048331)
Supplement: Text S3 — Multiple participants and animals. This describes what would be needed to extend the system to cater for multiple human and animal participants. (DOCX) [file pone.0048331.s003.docx]

Supporting Text S3

Beaming into the Rat World: Interaction Between Rat and Human Through an Immersive Virtual Environment

Jean-Marie Normand^1^, Maria V. Sanchez-Vives^2,3^, Christian Waechter^4^,
Elias Giannopoulos^1^, Bernhard Grosswindhager^5^, Bernhard Spanlang^1^,
Christoph Guger^5^, Gudrun Klinker^4^, Mandayam A. Srinivasan^6,7^, Mel Slater*^1,2,7^

^1^EVENT Lab, Faculty of Psychology, University of Barcelona, Spain

^2^Institució Catalana de Recerca i Estudis Avançats (ICREA), Barcelona, Spain

^3^Institut d’Investigacions Biomèdiques August Pi i Sunyer (IDIBAPS), Barcelona, Spain

^4^Fachbereich Informatik, Technische Universität München, Germany

^5^Guger Technologies (g.tec), Austria

^6^The Touch Lab, Research Laboratory of Electronics and Department of Mechanical Engineering, Massachusetts Institute of Technology, Cambridge, USA

^7^Department of Computer Science, University College London, UK

Corresponding Author:

*Mel Slater

ICREA (Institucio Catalana de Recerca i Estudis Avançats), Barcelona, Spain

melslater@ub.edu

# Multiple Participants and Animals

It would be relatively straightforward for this system to cater for multiple participants. Indeed, the only difference between the presented system and a multi-actor one would lie in the way data is exchanged between computers.

The tracking system could easily track multiple robots (each representing a different human participant) since it is based on a classical marker-tracking method. Hence with a different marker on top of each robot the tracking system would be able to track each of them without introducing much overhead. Tracking more than one rat could also be achieved with slight modifications to the current system. The Ubitrack system [1] used for the rat tracking in the experiment could be used to track up to 5 rats without impact on the performance. Of course some technical requirements would have to be enforced for multiple rat tracking, such as requiring the rats to be far from one another at the beginning of the tracking process in order to be able to detect each of them separately. A camera with a higher frame rate would also support the task of tracking multiple rats.

Additional problems that may arise if rats gather very close to one other could be handled by using individual particle filters in combination with our visual pattern matching for evaluation and a camera with a higher frame rate, such that positions during fast rat movements could be assigned better to the correct filter. However, in certain situations, when the rats crawl over each other, the tracking could fail.

For experiments designed to track multiple robots and animals at the same time (more than 5 robots and more than 5 animals), it would be safer to dedicate a single computer to the tracking system. This would ensure the tracking system would have enough computational power and help to prevent in the system lag.

Concerning the video stream, adding participants would also be easy to implement. The current application would require one stream per participant taking part in the experiment.

Additionally, a peer architecture could be implemented in order to simplify the integration of an unknown number of participants in the experiment. The XVR framework we used in this experiment offers such a possibility (see [2] for an example of XVR used in such a configuration) and could be used to stream both the video stream and the tracking data (positions of the participants and the rats).

Each new participant would be represented as a new peer connecting to a centralised server. Each peer could be of a different type (i.e. ‘animal peer’, ‘human peer’, ‘tracking peer’ or ‘video peer’) and would send data directly to the server. The server would have to dispatch data according to each peer’s role in the experiment, for example, sending the positions of the animals to the ‘human peers’ or sending the video stream to every ‘human peer’.

Of course, a multiple participant setup would require more computational power and additional computers might be required to perform such an experiment. This would be especially true for robot control, which requires the MATLAB software as well as Bluetooth connections (via dongles).

# References

1. Pustka D, Huber M, Waechter C, Echtler F, Keitler P, et al. (2011) Automatic configuration of Pervasive Sensor Networks for Augmented Reality. IEEE Pervasive Computing 10: 68-79.

2. Normand J-M, Spanlang B, Tecchia F, Carrozzino M, Swapp D, et al. (2012) Full Body Acting Rehearsal in a Networked Virtual Environment - A Case Study. PRESENCE - Teleoperators and Virtual Environments 21: 229-243.
