# Supplementary material for: Beaming into the Rat World: Enabling Real-Time Interaction between Rat and Human Each at Their Own Scale
Source: PLoS One. 2012 Oct 31;7(10):e48331. doi: 10.1371/journal.pone.0048331 (PMC3485138; doi:10.1371/journal.pone.0048331)
Supplement: Text S4 — Distance distributions in trials 1 and 2. This presents further analysis of the distances between the rat and human participants, and in particular there is a comparison between trials 1 and 2. (DOCX) [file pone.0048331.s004.docx]

Supporting Text S4

Beaming into the Rat World: Interaction Between Rat and Human Through an Immersive Virtual Environment

Jean-Marie Normand^1^, Maria V. Sanchez-Vives^2,3^, Christian Waechter^4^,
Elias Giannopoulos^1^, Bernhard Grosswindhager^5^, Bernhard Spanlang^1^,
Christoph Guger^5^, Gudrun Klinker^4^, Mandayam A. Srinivasan^6,7^, Mel Slater*^1,2,7^

^1^EVENT Lab, Faculty of Psychology, University of Barcelona, Spain

^2^Institució Catalana de Recerca i Estudis Avançats (ICREA), Barcelona, Spain

^3^Institut d’Investigacions Biomèdiques August Pi i Sunyer (IDIBAPS), Barcelona, Spain

^4^Fachbereich Informatik, Technische Universität München, Germany

^5^Guger Technologies (g.tec), Austria

^6^The Touch Lab, Research Laboratory of Electronics and Department of Mechanical Engineering, Massachusetts Institute of Technology, Cambridge, USA

^7^Department of Computer Science, University College London, UK

Corresponding Author:

*Mel Slater

ICREA (Institucio Catalana de Recerca i Estudis Avançats), Barcelona, Spain

melslater@ub.edu

# Distance Distributions in Trials 1 and 2

Consider the set of waveforms of distances between rat and robot for each participant and trial (for example, as shown in Figure 7 for one participant and trial). Of particular interest is the proportion of time (that is the proportion of samples) where the distance between rat and robot fell below a threshold, indicating a close approach, and whether this differed between the conditions of rat (trial 1) or supposed human opponent (trial 2). For a threshold between 0.2 m and 0.3 m the mean number of close encounters is lower in the human opponent condition (trial 2) than it is in the rat opponent condition.

Table S1 shows the means and standard deviations of the proportions of close encounters between the rat and robot by trial and rat. It is clear that there are no differences between the rats, but a probable difference between trials 1 and 2. A one-way within groups ANOVA shows that for a threshold of 0.2 m the difference between trials is significant (P = 0.03), also for 0.25 m (P = 0.018) and for 0.3 m (P = 0.048). Outside of these limits the significance level increases - for smaller thresholds because there were not many encounters smaller than the lower limit, and for greater thresholds because the distribution amongst the conditions is obviously more uniform.

**Table S1**

Mean and S.D. of Proportion of Close Encounters

Less Than the Given Threshold. Sampling over a period of 5 minutes, at a sampling rate of 0.2s as the unit of time

|  | Threshold < 0.2 m | | Threshold < 0.3 m | |
| --- | --- | --- | --- | --- |
|  | Opponent | | Opponent | |
|  | Rat (trial 1) | Human (trial 2) | Rat (trial 1) | Human (trial 2) |
| Rat A | 0.20 ± 0.11 | 0.16 ± 0.10 | 0.45 ± 0.13 | 0.38 ± 0.15 |
| Rat B | 0.18 ± 0.08 | 0.14 ± 0.09 | 0.43 ± 0.14 | 0.36 ± 0.18 |

The study of proxemics has shown that there are various acceptable levels of interpersonal distance between two people. Hall [1] refers to public (≥3.7 m, <7.6), social (≥1.2 m, <3.7), personal (≥0.46 m, <1.2), and intimate (<0.46 m) distances. There are implicit social norms that discourage a stranger entering into personal or intimate distance, except in special circumstances such as on a crowded train. It has been shown moreover that these distances are preserved in virtual environments in encounters between people and avatars [2,3]. Recall that the virtual space was 4 times larger than the arena, so that the arena distances of 0.2 m to 0.3 m represent virtual distances, at human scale of between 0.8 m and 1.2 m. These fall within the personal distance category, which would be in line with proxemics theory.

Now because of the experimental design where the human opponent condition was always second, it cannot be said that the above result is unambiguously due to the participant’s belief that the opponent was human, and was therefore conforming to rules of proxemics. However, there is additional evidence in favour of the interpretation that the result was due to proxemics.

**Table S2**

Rat Movement

Mean ± S.D. Distance Travelled (m) and Time Moving (s) over 5 minutes by Rat and Trial. Each cell is based on n = 9. The sampling unit of time is 0.2 s.

|  | Distance Travelled (m) | | Time Moving (s) | |
| --- | --- | --- | --- | --- |
|  | Rat Trial (1) | Human Trial (2) | Rat Trial (1) | Human Trial (2) |
| Rat A | 23.9 ± 6.4 | 19.3 ± 9.8 | 174 ± 38 | 150± 69 |
| Rat B | 20.8 ± 4.8 | 15.8 ± 6.9 | 168 ± 42 | 128 ± 60 |

**Table S3**

Human Participant Movement

Mean ± S.D. Distance Travelled (m) and Time Moving (s) in 5 minutes by Rat and Trial. Each cell is based on n = 9. The sampling unit of time is 0.2 s.

|  | Distance Travelled (m) | | Time Moving (s) | |
| --- | --- | --- | --- | --- |
|  | Rat Trial (1) | Human Trial (2) | Rat Trial (1) | Human Trial (2) |
| Rat A | 13.8 ± 1.9 | 14.7 ± 2.7 | 87 ± 10 | 92 ± 15 |
| Rat B | 11.9 ± 3.3 | 12.1 ± 2.6 | 79 ± 18 | 78 ± 17 |

Tables S2 and S3 suggest that the activity of the rat diminished in trial 2 but that the activity of the humans did not change (in fact may have increased slightly). ANOVA on Table S2 shows that the difference in mean distance between the trials is significant (P < 0.02) but the difference between the rats is not and there are no statistical interaction effects. The test was carried out on a log scale in order to avoid non-normally distributed residual errors. A Jarque-Bera test [4] does not reject the hypothesis of normality using the log scale (P > 0.5). On the average the rats tended to travel less in the second trial, which is consistent with the likelihood of having been satiated in the first trial. Table S2 also shows the mean amount of time that the rats were not stationary (up to a tolerance of 1 cm). Again there was a tendency for the rats to move less on the average in the second trial (P < 0.09, JB test does not reject normality, P > 0.15). (In what follows unless otherwise stated residual errors of tests are consistent with normality using the JB test).

Table S3 shows the equivalent data for the human movement. This is based on the tracked data for the robot device, and so is with respect to the same tracking system and timings as for the rats. ANOVA shows a significant difference between the rats with Rat B producing less movement in the participants than Rat A (P < 0.02 for distance, P < 0.04 for movement), but no other significant differences.

Table S3 suggests that the participants moved about the same in their second trial as in their first trial, in spite of the rats moving less in the second trial. It is therefore likely that the action was driven by the human participants, in which case there would have been no reason for their approach behaviour to change in the second trial compared with the first other than for their belief that their opponent was a human. Nevertheless, this interpretation should be treated with caution given the lack of counter-balanced design.

The reason for the choice of the second trial always being with the ‘human’ was a practical one. We discovered through many pilot trials that participants found it difficult to understand that they were interacting with a live rat. This is no doubt because the participants did not have the background to understand how this could operate technically, and also they were using a virtual reality system, and hence of course would expect *virtual* reality. We therefore adopted a set of procedures in order to overcome this, including switching views between the virtual scenario and live video streaming of the rat arena, while allowing participants to learn that as they moved themselves in the virtual environment they would also see that the robot had moved in the arena, and also that the spatial relationship between the rat and robot was the same as that between themselves and the human character in the virtual reality (see Supporting Video S1). We also adopted an additional procedure to make absolutely sure that the participants would understand that the whole setup was live (Supporting Text S1). Having eventually learned how to convince participants that the scenario was a live one, and that they were interacting with a remote and real rat, we did not have the technical capability to do the same with a remote human, including the requirement for the participant to be represented by a human-sized remote robot. However, given that participants did accept that they had interacted with a live rat, it was then easy to also convince them that they were later interacting with a live human - through the technique of briefly showing a short video of a human waiving at them, standing next to a small humanoid robot. This video was represented to them as ‘live’ in the same way that the truly live rat video streaming had been seen earlier. We are building the technical capability so that in future experiments it would be possible to counter-balance trials of remote animals and remote humans in order to study this issue further.

In any case we do not want to overemphasise the proxemics issue, since it should be recalled that the real point of the study was to see whether it was possible in such a set up for there to be a successful and rule-based interaction, and overall the results suggest that this is the case.

# References

1. Hall ET (1973) The hidden dimension. Leonardo: 94-94.

2. Bailenson J, Blascovich J, Beall A, Loomis J (2003) Interpersonal Distance in Immersive Virtual Environments. Personality and Social Psychology Bulletin 29: 819-833.

3. Llobera J, Spanlang B, Ruffini G, Slater M (2010) Proxemics with Multiple Dynamic Characters in an Immersive Virtual Environment ACM Transactions on Applied Perception (TAP) 8: Article 3.

4. Jarque CM, Bera AK (1980) Efficient tests for normality, homoscedasticity and serial independence of regression residuals. Economics Letters 6: 255-259.
